# The BMP7-Derived Peptide p[63-82] Reduces Cartilage Degeneration in the Rat ACLT–pMMx Model for Posttraumatic Osteoarthritis

**DOI:** 10.1177/19476035241233659

**Published:** 2024-03-19

**Authors:** Ellen G.J. Ripmeester, Jessica S.J.J. Steijns, Karolina A.P. Wijnands, Roderick H.M.J. Stassen, Vasek Pitelka, Laura C.W. Peeters, Andy Cremers, Nzekui M. S. A. Astryde, Alzbeta Chabronova, Don A.M. Surtel, Pieter J. Emans, Guus G.H. van den Akker, Bert van Rietbergen, Lodewijk W. van Rhijn, Marjolein M.J. Caron, Tim J.M. Welting

**Affiliations:** 1 Laboratory for Experimental Orthopedics, Department of Orthopedic Surgery, Maastricht University, Maastricht, The Netherlands; 2 Department of Physiology and Pharmacology, University of Western Ontario, London, ON, Canada; 3 Laboratory for Experimental Orthopedics, Department of Orthopedic Surgery, Maastricht University Medical Center, Maastricht, The Netherlands; 4 Department of Biomedical Engineering, Orthopaedic Biomechanics, Eindhoven University of Technology, Eindhoven, The Netherlands

**Keywords:** articular cartilage, osteoarthritis, anterior cruciate ligament tear—partial meniscectomy, peptides, subchondral bone, cartilage degeneration, pain

## Abstract

**Objective:**

Osteoarthritis (OA) is characterized by articular cartilage erosion, pathological subchondral bone changes, and signs of synovial inflammation and pain. We previously identified p[63-82], a bone morphogenetic protein 7 (BMP7)-derived bioactive peptide that attenuates structural cartilage degeneration in the rat medial meniscal tear-model for posttraumatic OA. This study aimed to evaluate the cartilage erosion-attenuating activity of p[63-82] in a different preclinical model for OA (anterior cruciate ligament transection—partial medial meniscectomy [anterior cruciate ligament transection (ACLT)-pMMx]). The disease-modifying action of the p[63-82] was followed-up in this model for 5 and 10 weeks.

**Design:**

Skeletally mature male Lewis rats underwent ACLT-pMMx surgery. Rats received weekly intra-articular injections with either saline or 500 ng p[63-82]. Five and 10 weeks postsurgery, rats were sacrificed, and subchondral bone characteristics were determined using microcomputed tomography (µCT). Histopathological evaluation of cartilage degradation and Osteoarthritis Research Society International (OARSI)-scoring was performed following Safranin-O/Fast Green staining. Pain-related behavior was measured by incapacitance testing and footprint analysis.

**Results:**

Histopathological evaluation at 5 and 10 weeks postsurgery showed reduced cartilage degeneration and a significantly reduced OARSI score, whereas no significant changes in subchondral bone characteristics were found in the p[63-82]-treated rats compared to the saline-treated rats. ACLT-pMMx-induced imbalance of static weightbearing capacity in the p[63-82] group was significantly improved compared to the saline-treated rats at weeks 5 postsurgery. Footprint analysis scores in the p[63-82]-treated rats demonstrated improvement at week 10 postsurgery.

**Conclusions:**

Weekly intra-articular injections of p[63-82] in the rat ACLT-pMMx posttraumatic OA model resulted in reduced degenerative cartilage changes and induced functional improvement in static weightbearing capacity during follow-up.

## Introduction

Osteoarthritis (OA) is the most prevalent joint disorder worldwide and leads to pathological functional impairment of the musculoskeletal apparatus. OA is characterized by erosion of the articular cartilage, pathological subchondral bone changes,^1,2^ signs of synovial inflammation,^3^ and joint pain.^4,5^ Patients with painful radiographic OA have a higher risk of dying prematurely,^6^ and their inactive and sedentary lifestyle, which is a consequence of OA pain and disabilities, contributes to the development of many other threatening chronic diseases, such as coronary heart disease and diabetes.^4^

There is currently no clinically approved drug-based therapy to influence the progression of OA, and treatment mainly aims to postpone invasive orthopaedic surgery to replace the affected joint with an artificial biomechanical implant, such as a total joint or hip prosthesis.^7^ The lack of drug-based therapies calls for an urgent need for developing disease-modifying OA drugs (DMOADs) that can slow down or halt the progression of OA on a structural and functional basis. Instead of treating the disease relatively downstream at the symptomatic level,^7^ treating OA at the cellular level is expected to provide an avenue for effective disease modification. In this respect, we have previously identified bioactive peptides derived from bone morphogenetic protein 7 (BMP7) that have the capacity to attenuate the OA-associated pathological chondrocyte phenotype and reduce the severity of structural cartilage damage in the rat medial meniscal tear (MMT)-model for posttraumatic OA (ptOA).^8,9^ The use of peptises is promising, as the biomolecular synthesis of short linear peptides is relatively straightforward. In general, peptides are less susceptible to conformational inactivation as compared to recombinant growth factors and can be formulated for sustained release from a drug carrier.^10^ It is understood that the identification of the disease-modifying activity of any drug in multiple preclinical models for the disease under study will improve the clinical translatability of preclinical data. In addition, anterior cruciate ligament (ACL)-related biomechanical instability of the knee joint is an important parameter in the development of knee OA,^11,12^ and therefore, a relevant condition to take into consideration when testing novel DMOAD candidates *in vivo*. In our previous study, we studied the p[63-82] peptide in the MMT model, while this study broadens the scope by combining a partial medial meniscectomy (pMMx) with an ACL injury. We hypothesize that frequent intra-articular (IA) administration of p[63-82] is able to structurally and functionally attenuate the course of OA longitudinal development in the rat anterior cruciate ligament transection (ACLT)-pMMx model for ptOA.^8^ To evaluate the OA development longitudinally, the cartilage erosion-attenuating activity of p[63-82] was determined at 5 and 10 weeks post-ACLT-pMMx surgery. In addition, potential disease-modifying activity in the subchondral bone compartment of the knee joint, as well as uncovering any p[63-82]-dependent functional improvement in weightbearing and gait were evaluated.

## Methods

### Animals

To investigate the potential of p[63-82] on OA disease modification, skeletally mature male Lewis rats (Charles River Laboratories, Sulzfeld, Germany) with a mean age of 3 months and an average weight of 360.6 ± 14.2 g were obtained. Before the start of the study, all rats were housed in groups (maximum of 3 rats per cage) during a 2-week acclimatization period. Throughout the study, rats were housed with two animals per cage and kept in a ventilated room with controlled temperature (19°C-24°C) and humidity (45%-65%) at the Central Animal Facilities of Maastricht University. Rats were subjected to a 12 hour light–dark cycle, received standard lab chow and water *ad libitum* throughout the experiment. Animal well-being and weight was monitored daily after surgery and from day 7 onwards weekly. Prior to surgery, rats were randomized to either saline as the vehicle control group or p[63-82] as the treatment group. Sample size was calculated according to the formula of L. Sachs *n* = (sigma/delta)2 × 15.7) and corrected for potential drop-out, resulting in seven animals per group for the 5-week follow-up and 10 animals per group for the 10-week follow-up. Before euthanisation via cardiac puncture, anesthesia was induced with 4% (v/v) and maintained with 2% (v/v) Isoflurane (Abbott Laboratories, Lake Bluff, Illinois, USA). The Maastricht University Animal Ethics Committee approved all experimental procedures prior to the study initiation (WP2018-004-001). The experiment was performed in accordance with all relevant guidelines and regulations (ARRIVE).

### Surgical Procedure

Rats were weighted and premedicated with 0.01 to 0.5 mg/kg body weight Buprenorphine (Bupaq; Richter Pharma AG, Wels, Austria) subcutaneously, after which anesthesia was induced with 4% Isoflurane. During surgery, anesthesia was maintained with 2% Isoflurane. Throughout the procedure, body temperature was continuously controlled and automatically regulated at a temperature of 36.5°C. Fluid resuscitation was provided before surgical intervention with a single subcutaneous warm sterile 0.9% saline injection (1.5 mL) (B. Braun Medical, Oss, the Netherlands). The right knee and surrounding area was shaved, and the surgical area was disinfected with betadine solution (100 mg/mL povidone-iodine (Meda, Mérignac, France). Just anterior to the medial collateral ligament, a skin incision was made to gain access to the joint capsule. The joint capsule was incised just medial of the patella tendon. Following the joint capsule opening, an ACLT and a pMMx were performed. Hemostasis was applied if necessary. The joint capsule was continuously sutured using dissolvable 5-0 Vicryl sutures (Ethicon, Bridgewater, New Jersey, USA). The skin was closed using interrupted dissolvable 4-0 Vicryl sutures (Ethicon). Buprenorphine (0.01-0.5 mg/kg body weight) was subcutaneously administered for postoperative analgesia for 48 hours.

Following surgery, body weight loss was not significantly different between the treatment groups (Suppl. Fig. S1). One animal (saline group, 10 week follow-up) was taken out of the experiment, due to its failure to recover in body weight, resulting in a humane endpoint during the study period.

### Blinding

The investigators performing the IA injections and different measurements including footprint analysis, static weightbearing, radiography and microcomputed tomography (µCT), sectioning, and histopathology (including scoring) were all blinded for the different treatment groups to which the rats were allocated.

### IA Injections of p[63-82]

Peptide design, synthesis, purification, and identication were performed as described before.^9^ The investigator responsible for preparing the injections was blinded for the identity of the p[63-82] and saline stock solutions. IA injections with sterile saline (0.9% NaCl), or p[63-82] (500 ng in 50 µl saline/injection/animal/week) were freshly diluted from the 10× concentrated peptide stock solution (final concentration p[63-82]: 10 ng/µl) in sterile saline. Under general anesthesia, the right knee and the surrounding area were shaved, and the injection site was disinfected with a betadine solution. Rats were injected intra-articularly through the patella tendon and left to recover. IA injections were given weekly, starting on day 6 postsurgery.

### Static Weightbearing

Measurements of weightbearing capacity deficits between the operated (right) and contralateral control (left) hindlimb of the rat were performed during a stationary position using an Incapacitance Tester (Linton Instrumentation, Norfolk, UK).^13^ Prior to the start of the experimental phase, rats were trained for 2 weeks, and baseline weightbearing measurements were obtained. All measurements were conducted during the 12 hour light phase. During the measurements, each rat was habituated to a static position in a restrainer box for 3 seconds using an external stimulus. Changes in weightbearing distribution between the left contralateral control and the operated right hindlimb were indicated as a percentage of total weightbearing by calculating the weight put on the operated hindlimb. Per measurement, an average of the three separate measurements per rat was used for statistical analysis.

### Footprint Analysis

Footprint analysis was performed to measure animal mobility presurgery and postsurgery. To assess the footprint pattern, ink was applied to the rear feet of rats prior to allowing the rats to walk on paper to obtain inked pawprints. The rats were allowed to run on paper through a 50-cm long polymethyl methacrylate (PMMA) track with a dark chamber at the end of the track. Prints obtained in motion were scored according to the scoring system previously described by Kumar *et al.*^14^ The footprint analysis score was determined as the average score of three individual scorers.

### Radiography and µCT

After sacrifice, preparated operated right knee joints (all rats) and contralateral control joints (nonoperated left joint of the saline group) were fixated for 4 days in 10% neutral-buffered formalin (final concentration 3.7% formaldehyde in 1× phosphate-buffered saline [PBS]; VWR; Radnor; Pennsylvania; USA) and washed in 1× PBS overnight. Knees were scanned in a micro-CT scanner (µCT 100, Scanco Medical, Brüttisellen, Switzerland). The knee joints were scanned in a closed holder at a resolution of 10 µm, with source energy of 70 kVp, intensity of 200 µA and an integration time of 300 ms. µCT image processing included Gauss filtering with σ = 0.8, support of 1 voxel and a voxel size of 10 µm, as well as segmentation of the bone phase using a global threshold of 220 per mile of the maximum gray value, corresponding to 456 mg HA/ccm. Contours were drawn semiautomatically to determine the volume of interest (VOI) of the subchondral bone of the tibia plateau. From the segmented images, the following morphology indices were determined: tissue volume (TV), bone volume (BV), subchondral porosity (Sc.Po = 1 − BV/TV), volumetric bone mineral density (vBMD), subchondral tissue mineral density (Sc.TMD), average subchondral thickness (Sc.Th.), and average pore diameter (Sc.Po.Dm.). The Sc.Th and Sc.Po.D. were determined using a 3D distance transformation algorithm.

### Histopathology

After µCT-scanning, knees were decalcified for 2 weeks in 10% formic acid (VWR) and washed in 1× PBS overnight. Before paraffin wax embedding, soft tissue, including the patella, was removed, and joints were cut into two equal halves along the medial collateral ligament and embedded with the cut planes facing down. Paraffin wax embedding started with the dehydration of the samples by leaving them overnight in 50% and 70% ethanol (EtOH; VWR), followed by subsequent overnight dehydration steps before placing the samples in paraffin. Serial frontal sections of 7 µm were cut with an RM2245 microtome (Leica Biosystems, Amsterdam, The Netherlands) spanning the loading portion of the knee joint as described by Gerwin *et al.*^8^ Sections were stained with intervals of five sections using Safranin-O/Fast Green staining according to standard protocols. Histological images were acquired with the M8 microscope and scanner (software version 2018-02-04; PreciPoint, Munich, Germany) with the UPlanSAPO 20× microscope objective (Olympus Cooperation, Tokyo, Japan) and analyzed with ViewPoint imaging software (version 1.0.0.9628; PreciPoint). For each rat, the most severely affected section was determined after which the two directly adjacent sections were additionally stained. These three resulting consecutive sections per rat were then scored for changes in joint integrity according to the Osteoarthritis Research Society International (OARSI) guidelines for cartilage and joint pathology scoring^8^ and the mean of these three scored sections per rat was used for statistical analysis.

### Statistical Analyses

Statistical significances were determined using GraphPad PRISM 5.0 (La Jolla, California, USA). Statistical significance was determined by a two-tailed Mann-Whitney *U* test. Specifics about the statistical tests are indicated in the corresponding figure legends. The statistical significance of all tests was set at *p* ≤ 0.05. Bars in graphs represent the mean ± standard error of the mean (SEM).

## Results

### Reduced Cartilage Degeneration in p[63-82] Treated Animals

To determine whether peptide p[63-82] has the potential to prevent or delay the progression of cartilage degeneration in the rat ACLT-pMMx model, we performed a microscopical histopathological evaluation of frontal sections of the knee joints of both treatment groups at 5 and 10 weeks post-ptOA induction.

**Figure 1.**
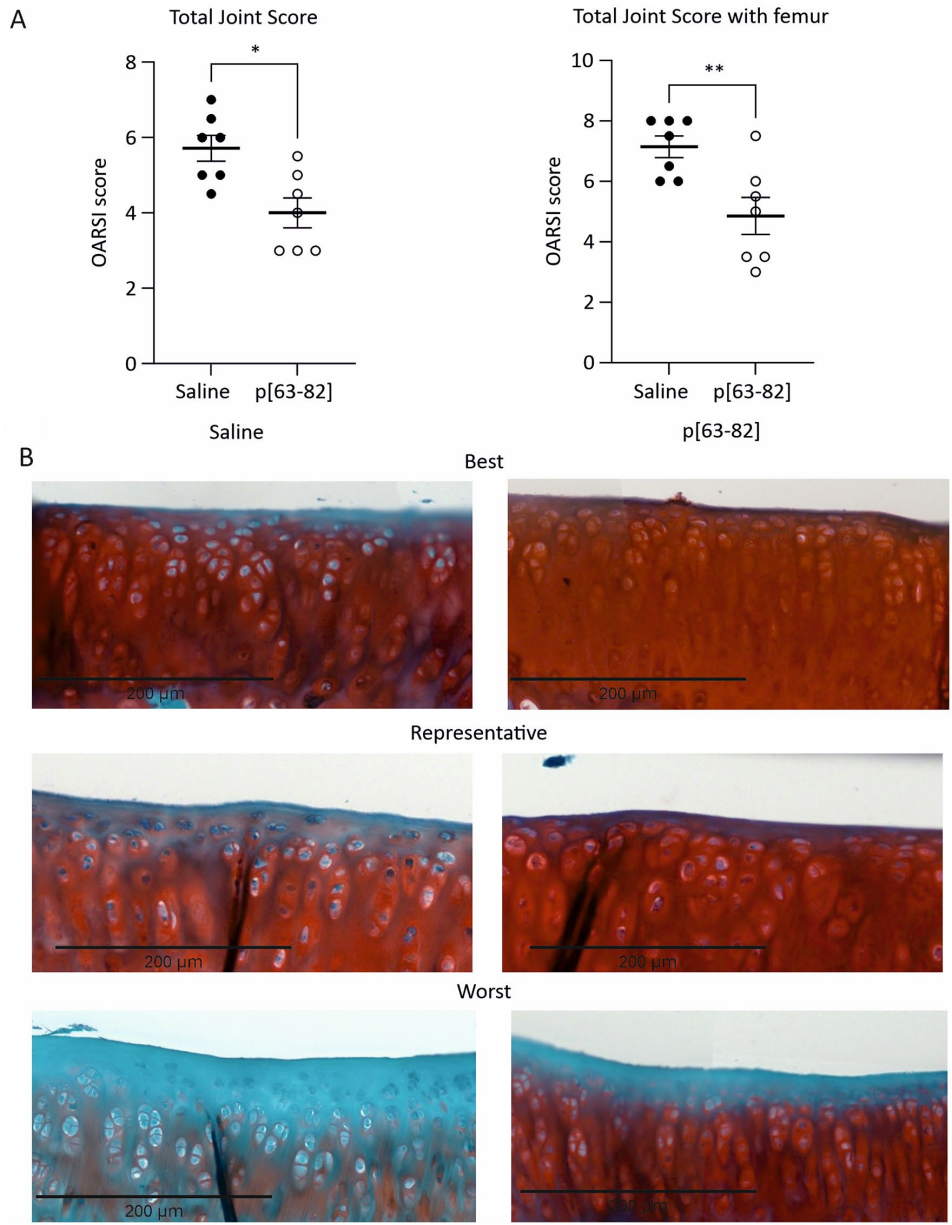
p[63-82] reduced cartilage degradation in the ACLT-pMMx animal model 5 weeks postsurgery. **A:** OARSI histological score for saline and p[63-82]-treated rats. OARSI histological score was determined according to the OARSI-scoring system per rat and represents an average of three separately scored sections per rat. Statistical significance was tested with an unpaired two-tailed Mann-Whitney *U* test. * is *p* < 0.05 compared to saline. Mean ± SEM. **B:** Medial tibial joint sections stained with Safranin-O/Fast Green. The best, representative and worst histological sections for saline (*n* = 7) or p[63-82] (*n* = 7) treated animals are shown in the panels.

Histopathology scoring at 5-week follow-up in the saline control group revealed degenerative changes in the medial tibia articular cartilage and to a lesser extent also in the medial femur articular cartilage (**Fig. 1A** and **B**, **Table 1**). OARSI total joint score with and without the femur component^8^ was statistically lower in the group receiving p[63−82] injections as compared to the saline-injected group. In saline-treated knee joints, the superficial zone across the tibial articular cartilage surface exhibited loss of Safranin-O staining (**Fig. 1B**) and the presence of enlarged (hypertrophic) chondrocytes. In the p[63-82] treated group, the superficial loss of Safranin-O staining was obvious, but fibrillation was rarely noticed and a lower incidence of enlarged chondrocytes was observed (**Fig. 1B**) compared to the saline-treated group. These observations were reflected in the OARSI histopathological subscores for medial tibia cartilage degeneration score, medial tibia cartilage degeneration width, and the medial tibia matrix loss width at 0% depth (**Table 1**), which were all significantly lower in the p[63-82] group as compared to the saline-treated group. In concert with this, the Safranin-O positivity of the femoral articular cartilage was generally lower in the saline group than in the p[63-82] treated group (**Fig. 1B**). However, the medial femur cartilage degeneration score was not significantly different between the two groups. At 5 weeks, we observed no differential effect on osteophyte formation with p[63-82] treatment compared to saline (**Table 1**). Together, these results demonstrate a protective effect on cartilage degeneration in the rat ACLT-pMMx model following weekly p[63-82] injections at 5 weeks follow-up.

**Table 1.** OARSI Histological Score for Saline and p[63-82]-Treated Rats 5 Weeks Postsurgery.

Parameter|Mean (SEM)		Saline (*n* = 7)	p[63-82] (*n* = 7)
**Medial Tibia Cartilage Degeneration Score**			
	Zone 1 (outside)	1.00 (0.15)	0.43 (0.20)
	Zone 2 (middle)	2.00 (0.18)	1.64 (0.28)
	Zone 3 (inside)	2.14 (0.28)	1.43 (0.35)
	**Total**	5.14 (0.18)	3.50 (0.39)**
**Medial tibia cartilage matrix loss width (µm)**			
	0% depth	438.0 (45.70)	295.4 (33.60)*
	50% depth	109.1 (15.10)	73.4 (18.60)
	100% depth	0.0 (0.00)	6.71 (6.70)
**Medial tibia depth ratio**			
	Total	0.24 (0.09)	0.23 (0.03)
**Tibia cartilage degeneration width (µm)**			
	Substantial	720.9 (50.2)	511.4 (50.00)*
	Total	1586.0 (45.7)	1232.0 (127.20)*
**Total Medial Tibia Bone Score**			
	Score	1.57 (0.17)	1.29 (0.15)
**Medial Femur Cartilage Degeneration Score**			
	Score	1.43 (0.17)	0.86 (0.24)
**Medial Tibia Osteophyte Score**			
	Score	0.57 (0.20)	0.50 (0.19)
**Total Joint Score**			
	Without femur	5.71 (0.34)	4.00 (0.39)*
	With femur	7.14 (0.36)	4.86 (0.61)*

OARSI histological score for saline and p[63-82]-treated rats. OARSI histological score was determined according to the OARSI-scoring system per rat and represents an average of three separately scored sections per rat. Statistical significance was tested with an unpaired two-tailed Mann-Whitney *U* test. * is *p* < 0.05 compared to saline. Mean ± SEM.

**Figure 2.**
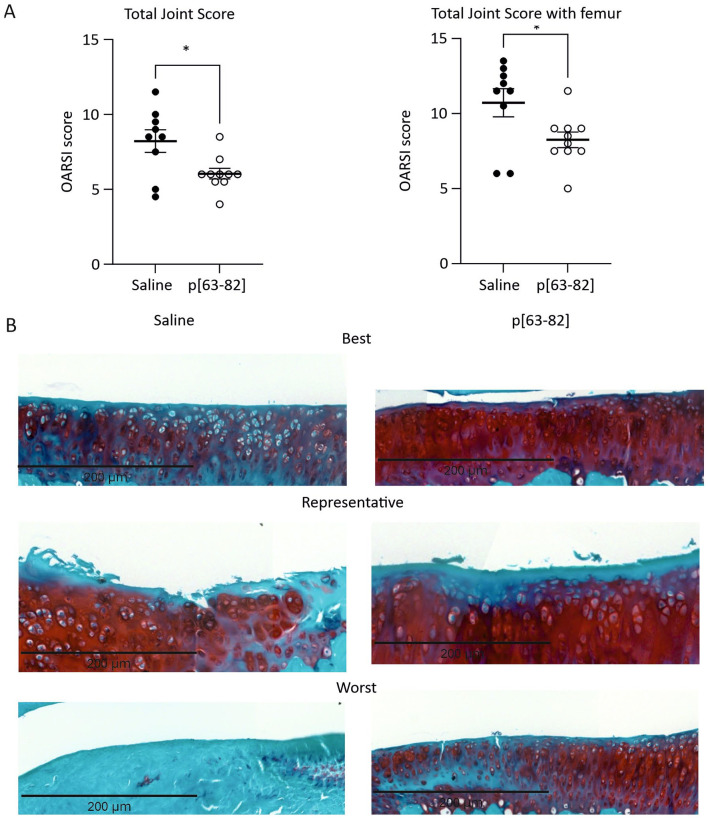
p[63-82] reduced cartilage degradation in the ACLT-pMMx animal model 10 weeks postsurgery. **A:** OARSI histological score for saline and p[63-82]-treated rats. OARSI histological score was determined according to the OARSI-scoring system per rat and represents an average of three separately scored sections per rat. Statistical significance was tested with an unpaired two-tailed Mann-Whitney *U* test. * is *p* < 0.05 compared to saline. Mean ± SEM. **B:** Medial tibial joint sections stained with Safranin-O/Fast Green. The best, representative, and worst histological sections for saline (*n* = 9) or p[63-82] (*n* = 10) treated animals are shown in the panels.

At 10-week follow-up, an increase in OARSI total joint score was observed in the saline control group, indicative of cumulative degenerative changes in the medial tibia articular cartilage (Suppl. Fig. S2). The OARSI total joint score with and without the femur component^8^ was at 10 weeks follow-up also statistically lower in the group that received the p[63−82] injections, as compared to the saline-injected group (**Fig. 2A** and **B**, **Table 2**). Similar to the 5-week follow-up, we observed significant improvements in the p[63-82]-treated group on medial tibia cartilage degeneration scores, matrix loss width at the superficial cartilage layer, and total medial tibial cartilage degeneration width (**Table 2**). In addition, the medial femur cartilage degeneration score and osteophyte score were now significantly higher in the control saline group as compared to the group receiving the peptide injections (**Table 2**). The p[63-82] peptide thus ameliorates articular cartilage degenerative changes in the rat ACLT-pMMx model for ptOA at both 5 and 10 weeks follow-up.

**Table 2.** OARSI Histological Score for Saline and p[63-82]-Treated Rats 10 Weeks Postsurgery.

Parameter|Mean (SEM)		Saline (*n* = 9)	p[63-82] (*n* = 10)
**Medial Tibia Cartilage Degeneration Score**			
	Zone 1 (outside)	1.94 (0.17)	1.95 (0.18)
	Zone 2 (middle)	2.44 (0.25)	2.10 (0.23)
	Zone 3 (inside)	2.43 (0.15)	1.75 (0.20)*
	**Total**	6.95 (0.37)	5.85 (0.31)*
**Medial tibia cartilage matrix loss width (µm)**			
	0% depth	1120.00 (121.10)	702.10 (59.40)*
	50% depth	482.10 (72.50)	381.70 (56.70)
	100% depth	93.70 (48.30)	18.15 (10.80)
**Medial tibia depth ratio**			
	Total	0.44 (0.11)	0.33 (0.06)
**Tibia cartilage degeneration width (µm)**			
	Substantial	643.30 (87.40)	562.20 (54.10)
	Total	1630.00 (79.60)	1399.00 (79.60)*
**Total Medial Tibia Bone Score**			
	Score	2.00 (0.39)	1.40 (0.22)
**Medial Femur Cartilage Degeneration Score**			
	Score	2.78 (0.15)	2.10 (0.22)*
**Medial Tibia Osteophyte Score**			
	Score	1.78 (0.37)	0.30 (0.17)**
**Total Joint Score**			
	Without femur	8.22 (0.76)	6.05 (0.36)*
	With femur	10.72 (0.94)	8.25 (0.52)*

OARSI histological score for saline and p[63-82]-treated rats. OARSI histological score was determined according to the OARSI-scoring system per rat and represents an average of three separately scored sections per rat. Statistical significance was tested with an unpaired 2-tailed Mann-Whitney U test.

* is *p* < 0.05 compared to saline. Mean ± SEM.

### P[63-82] Treatment Does not Alter Subchondral Bone Characteristics After ACLT-pMMx Surgery

**Figure 3.**
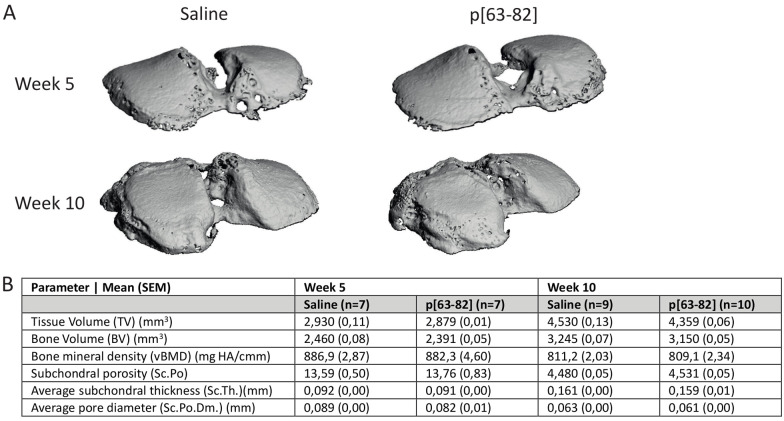
p[63-82] treatment does not alter subchondral bone changes induced by ACLT-pMMx surgery. Subchondral bone changes were assessed with µCT scanning. µCT scanning was conducted on the subchondral bone of the operated (*n* = 7 for 5-week follow-up groups and *n* = 9 for saline and *n* = 10 for peptide treatment group in the 10-week follow-up) tibia. **A:** Representative 3D µCT images of the tibia plateau from each experimental group. **B:** To determine subchondral bone changes, multiple markers were measured: tissue volume (TV), bone volume (BV), volumetric bone mineral density (vBMD), subchondral bone porosity (Sc.Po.), subchondral thickness (Sc.Th.), and subchondral porosity diameter (Sc.Po.Dm.). Statistical significance was tested with a two-tailed Mann-Whitney U test compared to saline. Mean ± SEM. No statistical differences were observed.

OA is characterized by erosion of the articular cartilage as well as pathological changes in the subchondral bone compartment,^1,2^ attributing to OA-related pain.^4,15,16^ To determine changes in the subchondral bone following ACLT-pMMx surgery of knee joints of saline or p[63-82]-treated rats, 3D µCT images of the subchondral tibia plateau were obtained. Following ACLT-pMMx surgery, multiple subchondral bone morphological parameters were significantly altered compared to contralateral control joints at both 5- and 10-week follow-ups. ACLT-pMMx surgery lead to an increase in mineralized TV, Sc.Po, and Sc.Po.Dm. While Sc.Th., vBMD, and Sc.TMD were reduced, BV was not significantly different in operated knee joints (Suppl. Fig. S3). No significant changes in all quantified bone characteristics measured by µCT were observed between saline and p[63-82] treatment (**Fig. 3A** and **B**). These results are also reflected in the not statistically different histological total medial tibia bone scores at 5 and 10 weeks in the saline and peptide-treated groups (**Tables 1** and **2**). In conclusion, while ACLT-pMMx surgery induced significant changes in the tibia plateau subchondral bone compartment, the observed subchondral bone changes were not significantly altered after p[63-82] treatment at both 5 and 10 weeks follow-up.

### Improvement of Pain-Related Behavior After p[63-82] Treatment

To determine functional alterations in pain-related behavior, we compared the static and dynamic weightbearing capacity deficits of the p[63-82]-treated animals with the saline-treated control group.^17-19^

**Figure 4.**
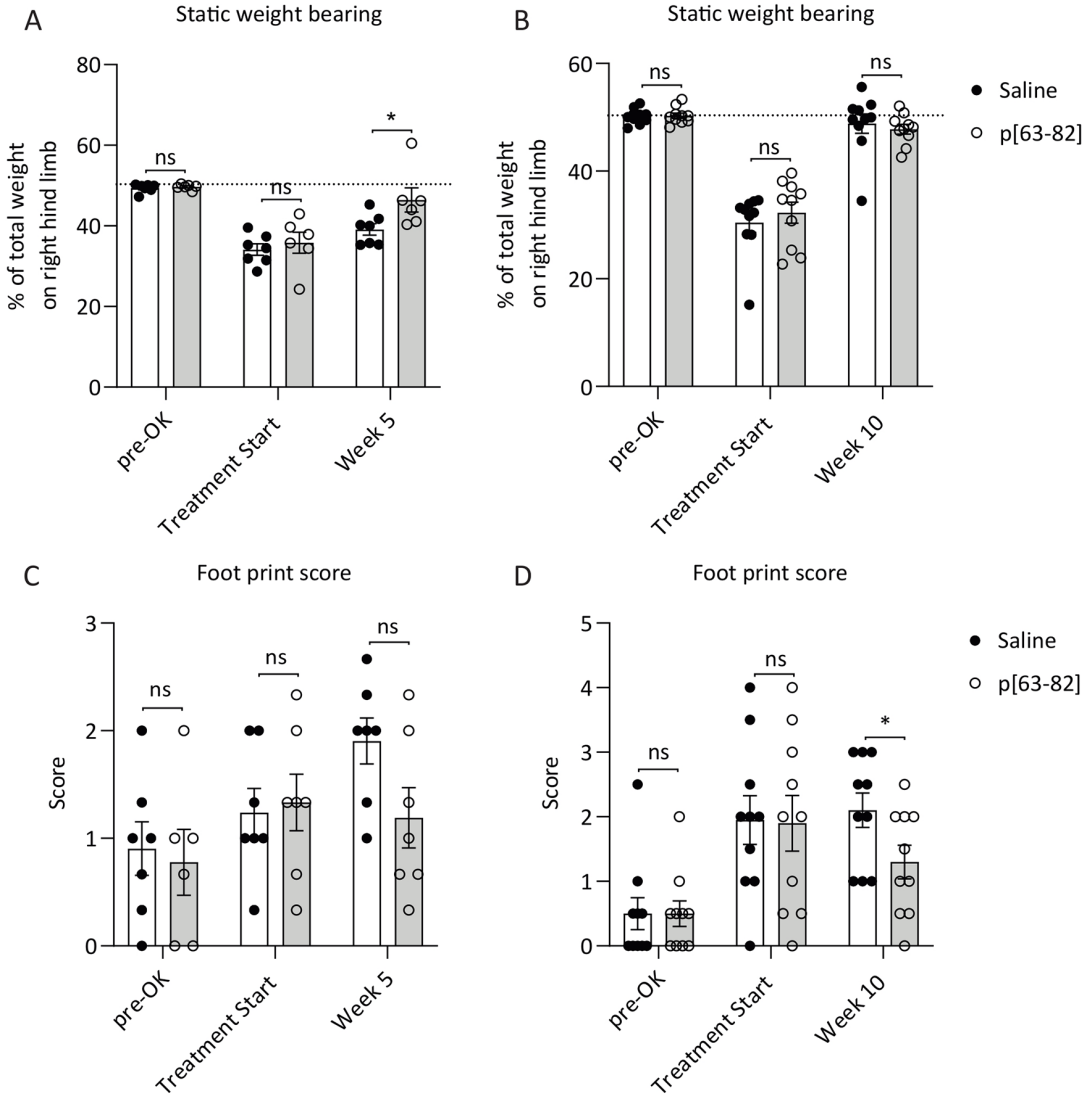
p[63-82] treatment in the ACLT-pMMx rat model decreases pain-related behavior. Static and dynamic weightbearing was measured weekly to assess pain-related behavior. **A:** Static weightbearing was measured with an incapacitance tester and is depicted as percent weightbearing on the operated hindlimb. Animals (*n* = 7 saline and *n* = 7 p[63-82] treatment) were followed up for 5 weeks postsurgery. **B:** Static weightbearing was measured with an incapacitance tester and is depicted as percent weightbearing on the operated hindlimb. Animals (*n* = 9 saline and *n* = 10 p[63-82] treatment) were followed up for 10 weeks postsurgery. **C:** Footprint analysis of rats 5 weeks postsurgery (*n* = 7 rats per group). The footprint score is the average of the three individual scorers. **D:** Footprint analysis of rats 10 weeks postsurgery (*n* = 9 saline and *n* = 10 p[63-82] treatment). The footprint score is the average of the three individual scorers. Statistical significance was tested between treatment groups (graph) with a two-tailed Mann-Whitney U test * is *p* < 0.05, ** is *p* < 0.01. Mean ± SEM.

One week post-ACLT-pMMx surgery (at the moment of the first IA injection), a significant reduction in the static weightbearing capacity in the operated right knee joints compared to their contralateral left limbs was observed in both treatment groups (**Fig. 4A** and **B**). At 5 weeks, the reduction in static weightbearing capacity in the saline-treated control group remained significantly decreased compared to the corresponding pre-surgical weightbearing measurements (**Fig. 4A**, white bars). For the p[63-82] treatment group, the static weightbearing capacity at 5 weeks recovered to pre-OK level (**Fig. 4A**, gray bars), and this was significantly improved compared to the saline control group. At 10-week follow-up, static weightbearing in both saline and p[62-82]-treated groups was returned to pre-OK level (**Fig. 4B**).

Footprint analysis^14^ was conducted to determine whether we could detect p[63-82]-dependent differences in dynamic weightbearing capacity. Footprint score was significantly increased for the saline treatment group at 5 and 10 weeks follow-up, compared to presurgery footprint score measurements. This is indicative of impaired dynamic weightbearing (**Fig. 4C** and **D**, white bars). For the p[63-82] treatment group in the 5-week follow-up, the footprint scores were not statistically different compared to the footprint scores from the saline control group for all time points measured (**Fig. 4C**). For the 10-week follow-up groups (**Fig. 4D**), no statistical difference was observed between saline and p[63-82] treated groups in the pre-OK and start-of-treatment time points. However, at 10 weeks of follow-up, we observed a statistically significant improvement in footprint scores in the p[62-82] treatment group, compared to the saline control group (**Fig. 4D**). Together with the improved static weightbearing capacity, these results demonstrate that IA treatment with p[63-82] resulted in reduced pain-related behavior in rat ACLT-pMMx.

## Discussion

In our previous study, IA injections with p[63-82] attenuated cartilage degeneration in the rat MMT model, by altering downstream chondrocyte SMAD signaling, which we determined was NKX3-2-dependent (a well-known transcriptional regulator for chondrocyte hypertrophy).^9,20^ In this study, we aimed to investigate whether the protective effect of p[63-82] on cartilage degeneration could be translated to other preclinical models for OA. The rat ACLT-pMMx model is less progressive than the rat MMT model^8^ and involves an ACL-dependent biomechanical destabilization of the knee joint. Rupture of the ACL with or without a meniscectomy is a major risk factor for OA development in human patients.^12^ Our data show that various histopathological aspects of ACLT-pMMx-dependent cartilage degeneration are less affected in the p[63-82] treated animals as compared to treatment with saline at both 5 and 10 weeks post-ACLT-pMMx surgery. This led to an overall significant reduction of the OARSI total joint scores at both time points. A reduced loss of Safranin-O positivity in the superficial layer of the tibia plateau and femur condyle articular cartilage, as well as a lower incidence of enlarged (hypertrophic) chondrocytes demonstrates that p[63-82] has a protective effect on cartilage degeneration in this model. A similar p[63-82]-dependent protective effect on cartilage integrity was also observed in the rat MMT model.^9^ Since osteophyte formation is an OA-pathological process that takes time,^21^ the 10-week follow-up in this study allowed us to also determine the effect of the p[63-82] peptide on osteophyte formation. Indeed, reduced osteophyte formation as a result of p[63-82]-treatment became apperent at 10 weeks follow-up. We speculate that the hypertrophy-inhibiting action of the p[63-82] peptide^9^ is involved in its impact on osteophyte formation, taking into account the endochondral ossification that is centrally involved in OA-related osteophyte formation.^22^

Subchondral bone alterations are a hallmark of OA and are thought to initiate or contribute to cartilage degenerative changes.^1,21,23^ Indeed, µCT scanning of the subchondral bone at 5 and 10 weeks into rat ACLT-pMMx progression revealed clear signs of bone remodeling typical for OA (Suppl. Fig. S3). Treatment with p[63-82] did not lead to significant improvements in the subchondral bone characteristics. We speculate that this can be caused by the biomechanically unstable nature of the ACLT-pMMx model and its large impact on the subchondral bone biology to allow for the detection of disease-modification in this joint tissue compartment. Another potential explanation is that any effects of the peptide on the subchondral bone compartment may take longer to appear, or because the intra-articularly injected peptide does not reach the subchondral bone compartment.

This study for the first time addressed whether p[63-82] has the capacity to change ptOA functional outcomes. Several methods have been reported to acquire quantifiable measurements for OA pain-related behavior in small animal models.^17^ Here we measured static and dynamic weightbearing as quantifiable measures for pain-related behavior.^17,18^ The static weightbearing distribution, measured by incapacitance testing, clearly showed that treatment with p[63-82] led to an accelerated normalization of ACLT-pMMx-induced disturbance of weightbearing. However, using a fairly generic method to assess gait, footprint measurements acquired from these rats demonstrated no statistically significant improvement in dynamic weightbearing in the p[63-82] group at 5 weeks follow-up. However, the footprint score of the p[63-82] treatment group was significantly improved at 10 weeks follow-up when compared to the saline treatment group. Pain in OA has been attributed to various aspects of its pathobiology. OA-related changes in the subchondral bone are associated with pain-related behavior in human patients and rat models.^15,16^ In humans, OA pain has been associated with synovitis^24^ and damage-associated molecular patterns (DAMPs) generated during the course of OA are thought to trigger pain mechanisms.^25,26^ While we did not detect p[63-82]-related significant differences in synovitis (Suppl. Fig. S4) or subchondral bone characteristics, we speculate that the reduced magnitude of cartilage degeneration induced by p[63-82] may lead to a reduction of DAMPs originating from degenerating cartilage.^27^ This may lead to a less severe pain sensation (as measured by the footprint analysis and incapacitance testing), although this hypothesis requires further investigation in the future. In addition, we previously discovered that p[63-82] can reduce the expression of interleukin-6 (IL6) and cyclo-oxygenase 2 in human articular chondrocytes, and attenuates the release of prostaglandin E2 (PGE_2_) from human OA cartilage, infrapatellar fat pad and meniscus tissue.^9^ IL6 and PGE_2_ are nociceptive stimuli in OA pain.^28^ IL6 in OA synovial fluid has been correlated with pain^29^ and PGE_2_ sensitizes dorsal root ganglia neurons in OA.^30^ We therefore speculate that the p[63-82]-dependent improvement in static weightbearing deficits observed in our ACLT-pMMx model may originate from a reduction of IA IL6 and PGE_2_ synthesis by the articular cartilage. Alternatively, preservation of biomechanically more favorable articular cartilage by p[63-82] may also have played a role in the observed improvement of static weightbearing distribution.

The dosing and IA administration frequency of p[63-82] were different between the current ACLT-pMMx study (once a week 500 ng) and our previous MMT study (twice a week 100 ng). In both cases, this is in the same dose range as studies reporting on full-length BMP7 efficacy in rat models,^31,32^ but much lower than rat studies reporting on OA disease-modifying activity of the REG-O3 peptide in the rat ACLT-pMMx model.^33^ In order to fully uncover the disease-modifying potency of p[63-82], further studies are needed to determine optimal dosing, formulation, and administration frequency. The long-term efficacy and effects need to be studied in preclinical models that resemble the anatomy of the human knee and the age-related and chronic nature of human OA in greater detail.^34-36^

In conclusion, this study shows that weekly IA injections of p[63-82] in the rat ACLT-pMMx ptOA model resulted in a functional improvement in static weightbearing capacity during follow-up, and specifically reduced tissue degenerative changes in the articular cartilage layer. This strengthens our previously conducted study^9^ enhancing the clinical translatability of p[63-82].

## Supplemental Material

sj-tif-1-car-10.1177_19476035241233659 – Supplemental material for The BMP7-Derived Peptide p[63-82] Reduces Cartilage Degeneration in the Rat ACLT–pMMx Model for Posttraumatic Osteoarthritis Supplemental material, sj-tif-1-car-10.1177_19476035241233659 for The BMP7-Derived Peptide p[63-82] Reduces Cartilage Degeneration in the Rat ACLT–pMMx Model for Posttraumatic Osteoarthritis by Ellen G.J. Ripmeester, Jessica S.J.J. Steijns, Karolina A.P. Wijnands, Roderick H.M.J. Stassen, Vasek Pitelka, Laura C.W. Peeters, Andy Cremers, Nzekui M. S. A. Astryde, Alzbeta Chabronova, Don A.M. Surtel, Pieter J. Emans, Guus G.H. van den Akker, Bert van Rietbergen, Lodewijk W. van Rhijn, Marjolein M.J. Caron and Tim J.M. Welting in CARTILAGE

sj-tif-2-car-10.1177_19476035241233659 – Supplemental material for The BMP7-Derived Peptide p[63-82] Reduces Cartilage Degeneration in the Rat ACLT–pMMx Model for Posttraumatic Osteoarthritis Supplemental material, sj-tif-2-car-10.1177_19476035241233659 for The BMP7-Derived Peptide p[63-82] Reduces Cartilage Degeneration in the Rat ACLT–pMMx Model for Posttraumatic Osteoarthritis by Ellen G.J. Ripmeester, Jessica S.J.J. Steijns, Karolina A.P. Wijnands, Roderick H.M.J. Stassen, Vasek Pitelka, Laura C.W. Peeters, Andy Cremers, Nzekui M. S. A. Astryde, Alzbeta Chabronova, Don A.M. Surtel, Pieter J. Emans, Guus G.H. van den Akker, Bert van Rietbergen, Lodewijk W. van Rhijn, Marjolein M.J. Caron and Tim J.M. Welting in CARTILAGE

sj-tif-3-car-10.1177_19476035241233659 – Supplemental material for The BMP7-Derived Peptide p[63-82] Reduces Cartilage Degeneration in the Rat ACLT–pMMx Model for Posttraumatic Osteoarthritis Supplemental material, sj-tif-3-car-10.1177_19476035241233659 for The BMP7-Derived Peptide p[63-82] Reduces Cartilage Degeneration in the Rat ACLT–pMMx Model for Posttraumatic Osteoarthritis by Ellen G.J. Ripmeester, Jessica S.J.J. Steijns, Karolina A.P. Wijnands, Roderick H.M.J. Stassen, Vasek Pitelka, Laura C.W. Peeters, Andy Cremers, Nzekui M. S. A. Astryde, Alzbeta Chabronova, Don A.M. Surtel, Pieter J. Emans, Guus G.H. van den Akker, Bert van Rietbergen, Lodewijk W. van Rhijn, Marjolein M.J. Caron and Tim J.M. Welting in CARTILAGE

sj-tiff-4-car-10.1177_19476035241233659 – Supplemental material for The BMP7-Derived Peptide p[63-82] Reduces Cartilage Degeneration in the Rat ACLT–pMMx Model for Posttraumatic Osteoarthritis Supplemental material, sj-tiff-4-car-10.1177_19476035241233659 for The BMP7-Derived Peptide p[63-82] Reduces Cartilage Degeneration in the Rat ACLT–pMMx Model for Posttraumatic Osteoarthritis by Ellen G.J. Ripmeester, Jessica S.J.J. Steijns, Karolina A.P. Wijnands, Roderick H.M.J. Stassen, Vasek Pitelka, Laura C.W. Peeters, Andy Cremers, Nzekui M. S. A. Astryde, Alzbeta Chabronova, Don A.M. Surtel, Pieter J. Emans, Guus G.H. van den Akker, Bert van Rietbergen, Lodewijk W. van Rhijn, Marjolein M.J. Caron and Tim J.M. Welting in CARTILAGE
