# Supplementary figures and images for: The BMP7-Derived Peptide p[63-82] Reduces Cartilage Degeneration in the Rat ACLT–pMMx Model for Posttraumatic Osteoarthritis
Source: Cartilage. 2024 Mar 19;16(4):495–506. doi: 10.1177/19476035241233659 (PMC11569685; doi:10.1177/19476035241233659)

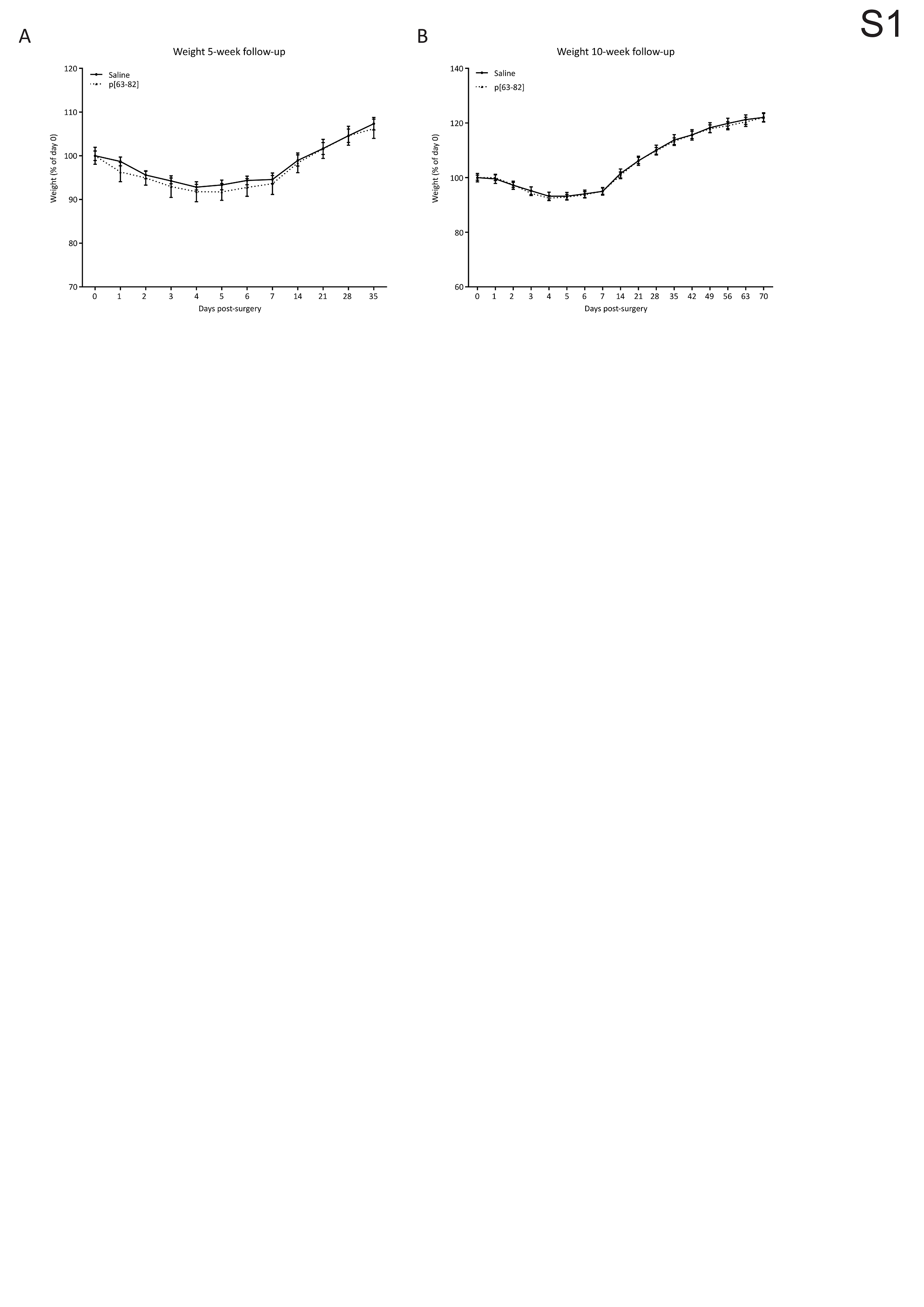

Supplement: sj-tif-1-car-10.1177_19476035241233659 – Supplemental material for The BMP7-Derived Peptide p[63-82] Reduces Cartilage Degeneration in the Rat ACLT–pMMx Model for Posttraumatic Osteoarthritis [file sj-tif-1-car-10.1177_19476035241233659.tif]

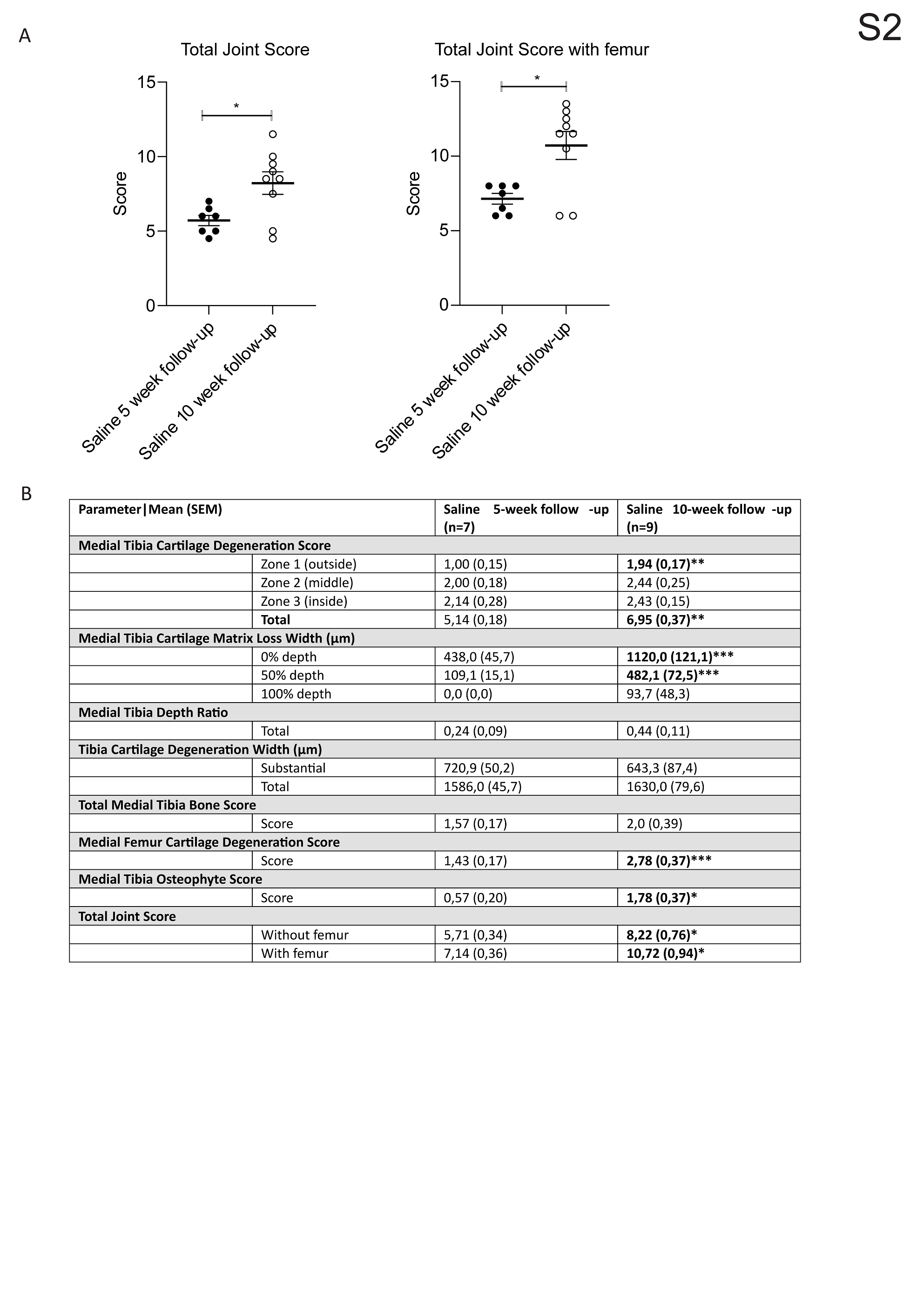

Supplement: sj-tif-2-car-10.1177_19476035241233659 – Supplemental material for The BMP7-Derived Peptide p[63-82] Reduces Cartilage Degeneration in the Rat ACLT–pMMx Model for Posttraumatic Osteoarthritis [file sj-tif-2-car-10.1177_19476035241233659.tif]

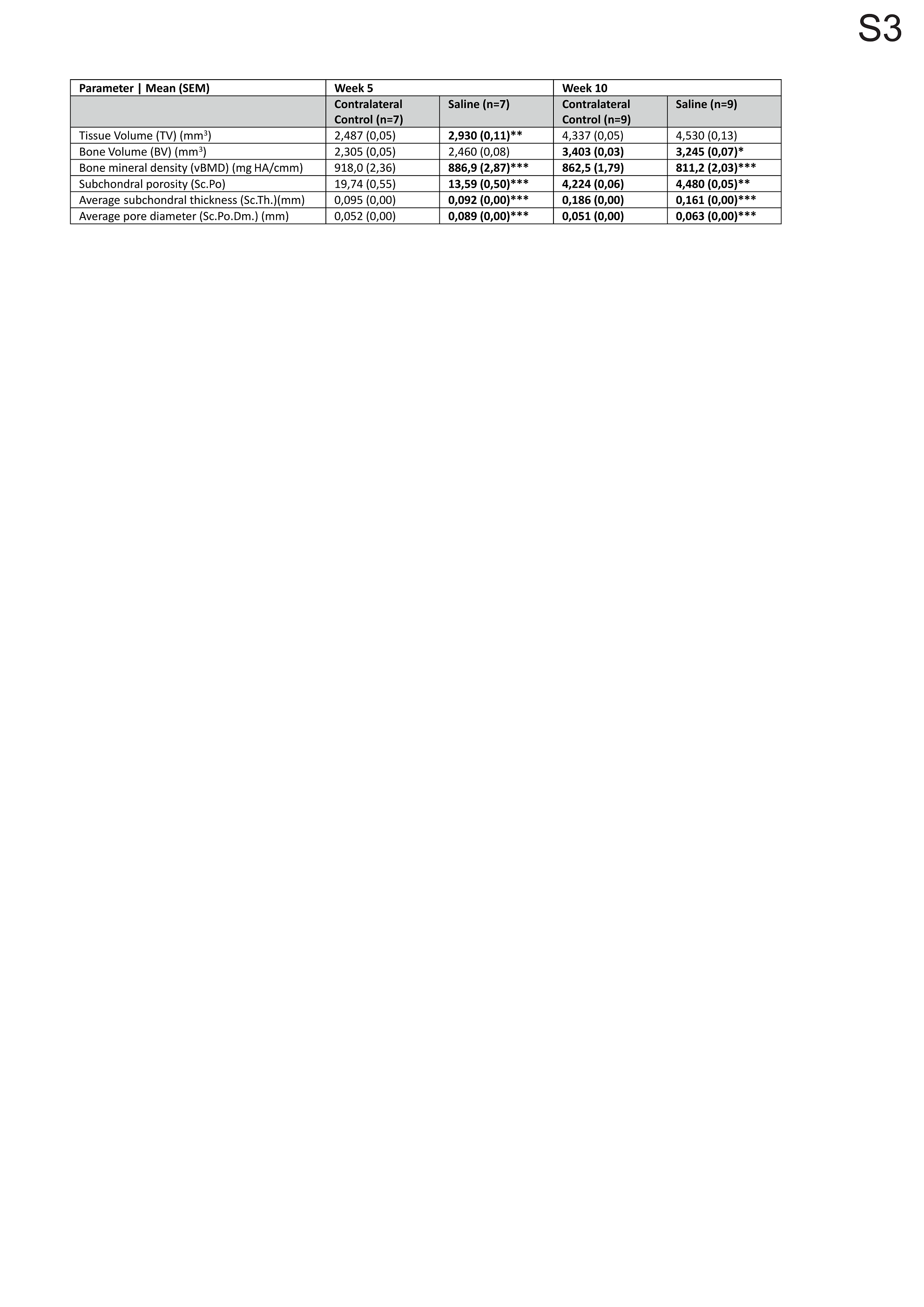

Supplement: sj-tif-3-car-10.1177_19476035241233659 – Supplemental material for The BMP7-Derived Peptide p[63-82] Reduces Cartilage Degeneration in the Rat ACLT–pMMx Model for Posttraumatic Osteoarthritis [file sj-tif-3-car-10.1177_19476035241233659.tif]

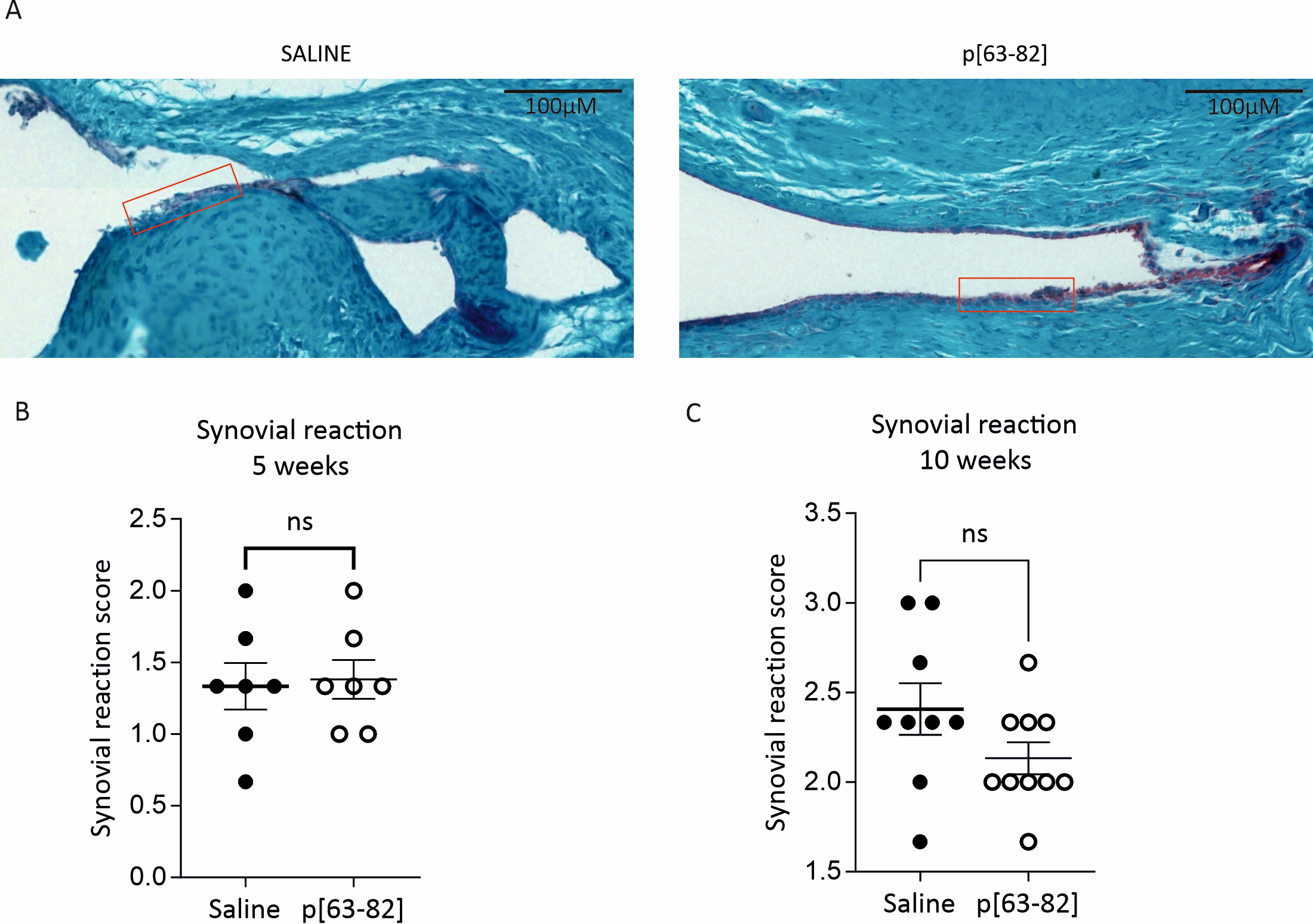

Supplement: sj-tiff-4-car-10.1177_19476035241233659 – Supplemental material for The BMP7-Derived Peptide p[63-82] Reduces Cartilage Degeneration in the Rat ACLT–pMMx Model for Posttraumatic Osteoarthritis [file sj-tiff-4-car-10.1177_19476035241233659.tiff]
